# Reduced resting‐state functional connectivity and sleep impairment in abstinent male alcohol‐dependent patients

**DOI:** 10.1002/hbm.24749

**Published:** 2019-08-04

**Authors:** Jingjing Liu, Wanye Cai, Meng Zhao, Wenlong Cai, Feng Sui, Wenbao Hou, Hongde Wang, Dahua Yu, Kai Yuan

**Affiliations:** ^1^ School of Life Science and Technology Xidian University Xi'an Shaanxi People's Republic of China; ^2^ Engineering Research Center of Molecular and Neuro Imaging Ministry of Education Xi'an People's Republic of China; ^3^ Xilinguole Meng Mongolian General Hospital Xilinhaote Inner Mongolian People's Republic of China; ^4^ Inner Mongolia Key Laboratory of Pattern Recognition and Intelligent Image Processing, School of Information Engineering Inner Mongolia University of Science and Technology Baotou Inner Mongolia People's Republic of China; ^5^ Guangxi Key Laboratory of Multi‐Source Information Mining and Security Guangxi Normal University Guilin People's Republic of China

**Keywords:** alcohol dependence, resting‐state functional connectivity, sleep impairment, thalamus

## Abstract

Alcohol dependence is associated with poor sleep quality, which has both been implicated with thalamocortical circuits function. To identify the possible roles of these circuits in the alcohol‐sleep association, we investigated the volume of both left and right thalamus and corresponding resting‐state functional connectivity (RSFC) differences between 15 alcohol‐dependent patients (AD) and 15 healthy controls (HC) male participants. The neuroimaging findings were then correlated with clinical variables, that is, Alcohol Use Disorders Identification Test (AUDIT) and Pittsburgh Sleep Quality Index (PSQI). Additionally, mediation analysis was carried out to test whether the thalamocortical RSFC mediates the relationship between drinking behavior and sleep impairments in AD when applicable. We observed a significant positive correlation between AUDIT score and PSQI score in AD. Compared with HC, AD showed reduced RSFC between the left thalamus and medial prefrontal cortex (mPFC), orbitofrontal cortex, anterior cingulate cortex (ACC), and right caudate. We also observed a negative correlation between RSFC of the left thalamus–mPFC and PSQI score in AD. More importantly, the left thalamus–mPFC RSFC strength mediated the relationship between AUDIT score and PSQI score in AD. No significant difference was detected in the normalized volume of both left and right thalamus, and volumes were not significantly correlated with clinical variables. Our results demonstrate that AD show abnormal interactions within thalamocortical circuits in association with drinking behaviors and sleep impairments. It is hoped that our study focusing on thalamocortical circuits could provide new information on potential novel therapeutic targets for treatment of sleep impairment in alcohol‐dependent patients.

## 1 INTRODUCTION

Alcohol dependence is a psychiatric disorder characterized by a compulsive drive toward alcohol consumption and inability to inhibit its consumption despite negative consequences. Individuals with alcohol dependence report poor sleep and disturbances of sleep (Luc et al., 2006) and the prevalence of sleep problems has been reported to be as high as 91% in alcohol‐dependent (AD) patients (Zhabenko, Wojnar, & Brower, 2012). For instance, high alcohol consumption is associated with decreases in rapid‐eye movement (REM) sleep, sleep continuity and sleep efficiency, and also increases in sleep latency (time to fall asleep) and sleep duration (Chaput, McNeil, Després, Bouchard, & Tremblay, 2012). These disturbances have caused serious impacts on the overall health and social well‐being of the individual. Therefore, it is of great significance to investigate the underlying neural mechanisms of sleep impairments in AD patients.

The important roles of the thalamus in alcohol dependence have been revealed by previous studies (Gilman, Smith, Ramchandani, Momenan, & Hommer, 2012; Hu, Ide, Zhang, Sinha, & Chiang‐shan, 2015; Schmaal et al., 2013; Zhornitsky et al., 2018), such as the implications of the modular dysfunction and structural deficits of thalamus in reward processing and cognitive control (Chanraud et al., 2007; Grodin & Momenan, 2017; Pitel, Segobin, Ritz, Eustache, & Beaunieux, 2015). Disruptions to these psychological processes are critical features of alcohol dependence, and the thalamus appears to be particularly vulnerable to the influence of alcohol (Pitel et al., 2015). Meanwhile, the thalamus plays crucial roles in sleep and circadian rhythmicity (Jan, Reiter, Wasdell, & Bax, 2009; Krone et al., 2017). It is worth noting that insomnia patients had lower waking metabolism and less thalamic activation than healthy controls (Nofzinger et al., 2006; O'Byrne, Berman Rosa, Gouin, & Dang‐Vu, 2014). The thalamus acts by inducing, maintaining, and advancing nonrapid eye movement (NREM) sleep assisted by melatonin which acts by promoting spindle formation (Jan et al., 2009).

Taken together, alcohol dependence is associated with significant sleep disturbances (Chakravorty, Chaudhary, & Brower, 2016), which are often accompanied by abnormal thalamus dysfunction (Li et al., 2018). The thalamus plays an essential role in the dorsal relay of the brainstem reticular activating system, controlling cortical activation that occurs during wakefulness and REM sleep (Thakkar, Sharma, & Sahota, 2015). The thalamus is not a unitary structure but is composed of several nuclei which cooperate to complete various functions through strong connections with neocortex via radiating fibers (Sherman, 2016). Thalamocortical function has been intimately tied to both addiction and sleep in prior research. For instance, cigarette smokers showed reduced connectivity between the thalamus and the dorsolateral PFC, and the strength of the dorsolateral PFC–thalamus connectivity was negatively associated with severity of nicotine dependence (Wang et al., 2017). Similar results were also shown in ketamine‐dependent individuals (Liao et al., 2016). In addition, thalamocortical neurons are essential for thalamocortical information transfer and regulation of arousal and sleep (Del Felice, Formaggio, Storti, Fiaschi, & Manganotti, 2012; Krone et al., 2017). These findings led us to hypothesize that thalamus centered RSFC mediates alcohol dependence and sleep impairment in AD patients. Therefore, the purposes of this study were to (a) assess the relationship between sleep quality (i.e., PSQI) and drinking behaviors in AD patients; (b) detect the volume and RSFC differences of thalamus between AD patients and controls and estimate their associations with drinking behaviors and sleep quality; (c) test the possible mediator role of the thalamus as the relationship between alcohol dependence and sleep impairment in drinkers. We hoped that by linking circuit‐level interactions between brain regions (especially the thalamus and PFC) to particular dimensions of behavioral dysfunction, we could shed new light on the neural associations of sleep impairment in AD patients.

## 2 MATERIALS AND METHODS

All recruitment and testing procedures were reviewed and approved by the ethics committee of medical research in Xilinguole Meng Mongolian General Hospital, Xilinhaote, Inner Mongolian, China. Experimental procedures of this study (psychometric interviews and MRI) were explained and all participants provided written informed consent to participate. Participants were compensated $50.

### 2.1 Participants

This study included 15 AD patients and 15 age‐ and gender‐matched healthy controls (HC) male participants. We instructed participants to have a good night's sleep and not to drink alcohol 48 hr before the study days. Inclusion criteria for AD patients were as follows: (a) ages 21–55 years; (b) current alcohol dependence met the Diagnostic and Statistical Manual of Mental Disorders, 4th edition (DSM‐IV); (c) no DSM‐IV axis I disorder or biomedical condition that may have adversely affected brain neurobiology; (d) no current or history of substance use disorder (other than alcohol dependence in AD group); and (e) participants enrolled in the current study were nonsmokers. Healthy controls were identified as those who showed negative test results for the diagnostic criteria of alcohol abuse in DSM‐IV. Participants in this study did not drink coffee at all, and so they were not instructed one way or the other. All of the participants were right‐handed as measured by the Edinburgh Handedness Inventory. Demographic data and study characteristics of the sample are shown in Table 1.

**Table 1:** Demographic characteristics of AD and HC in this study

Items	AD (*n* = 15)	HC (*n* = 15)	*t*	*p*‐value
Age (years)	47.3 ± 5.0	47.3 ± 4.9	0.00	1.00
BMI	21.3 ± 1.12	21.4 ± 1.4	0.30	.768
Education years	13.2 ± 2.6	14.7 ± 2.0	1.75	.091
ADS	21.5 ± 10.8	9.6 ± 5.3	3.83	**.001**
AUDIT	17.1 ± 10.0	6.3 ± 2.6	4.06	**<.001**
SAS	47.7 ± 16.9	35.7 ± 3.9	2.70	**.012**
SDS	49.2 ± 17.3	39.1 ± 4.6	2.18	**.038**
BIS‐11	66.0 ± 12.4	58.2 ± 8.7	2.00	.056
Drinks/week	15.8 ± 6.6	6.8 ± 2.3	5.05	**<.001**
Sleep duration	7.3 ± 0.9	7.0 ± 1.0	0.75	.463
PSQI	7.5 ± 3.9	4.9 ± 1.4	2.41	**.026**
A(subjective sleep quality)	1.27 ± 0.88	0.87 ± 0.35	1.63	.115
B (sleep latency)	1.20 ± 0.86	0.73 ± 0.46	1.85	.075
C (sleeping duration)	0.87 ± 0.83	0.73 ± 0.46	0.54	.591
D (habitual sleep efficiency)	0.87 ± 0.99	0.13 ± 0.35	2.7	**.012**
E (sleep disturbance)	1.60 ± 0.91	1.07 ± 0.45	2.03	.052
F (use of sleep medications)	0.13 ± 0.35	0.07 ± 0.26	0.59	.559
G (daytime dysfunction)	1.53 ± 1.06	1.20 ± 0.41	1.13	.266

Values are mean ± SD unless otherwise indicated. Bold values refer to the significant differences.

Exclusion criteria for both groups were (a) history of neurological or other physical diseases such as respiratory, cardiac, renal, hepatic, and endocrinal diseases; (b) any penetrating head trauma and closed head injury with loss of consciousness >10 min; (c) any medications currently that may affect brain functioning within 2 weeks; and (d) any current use of illicit substances, verified by toxicology screening.

The neuropsychiatric interview was performed by psychiatrists on the day of the fMRI scan. The sleep measurement of participants in this study was assessed by the self‐reported Pittsburgh Sleep Quality Index (PSQI) score, which combines seven component scores: Subjective sleep quality, sleep latency, sleep duration, habitual sleep efficiency, sleep disturbances, use of sleeping medication, and daytime dysfunction. The PSQI is a well‐validated 19‐item self‐report questionnaire that assesses sleep quality and quantity over the past month, which is considered to be a good measure of insomnia (Dietch et al., 2016). Scores range from 0 to 21 and higher scores represent worse sleep.

The measure of alcohol dependence severity was evaluated with the Alcohol Dependence Scale (ADS; Skinner & Allen, 1982); the frequency with which individuals engage in various indicators of alcohol abuse measured by the Alcohol Use Disorders Identification Test (AUDIT; Babor, Higgins‐Biddle, Saunders, & Monteiro, 2001). The AUDIT developed by the World Health Organization (WHO) as a self‐report screening test to identify severity of alcohol use disorders and provide an overall measure of hazardous drinking. Hazardous use, dependence symptoms, and harmful use were the three symptom areas covered by the 10‐item scale (Lundin, Hallgren, Balliu, & Forsell, 2015; World Health Organization, 2001). Total scores range from 0 to 40 and higher scores represent worse drinking. In addition, the participants also completed standardized questionnaires assessing depressive and anxiety symptomatology by Zung Self‐Rating Anxiety Scale (SAS; Zung, 1971) and Zung Self‐Rating Depression Scale (SDS; Zung, 1965). The impulsivity was measured by the Barratt Impulsiveness Scale (BIS‐11; Patton, Stanford, & Barratt, 1995).

### 2.2 MRI data acquisition

Functional imaging data were acquired using a 3T Philips scanner (Achieva; Philips Medical Systems, Best, The Netherlands). Prior to the MRI scanning, subjects were instructed to refrain from alcohol and other drugs consumption for the prior 48 hr, which was confirmed with a urine drug screen and a breath test for alcohol. For each participant, the high‐resolution three‐dimensional T1‐weighted image was acquired using a magnetization‐prepared rapid gradient echo (repetition time [TR] = 8.5 ms; echo time [TE] = 3.4 ms; data matrix = 240 × 240; slices = 140; field of view [FOV] = 240 × 240 mm^2^; flip angle [FA] = 12°; voxel size = 1 × 1 × 1 mm^3^). BOLD fMRI scans were obtained by using a multislice single‐shot gradient echo‐planar imaging (EPI) sequence with the following scan parameters: TR = 2000 ms; TE = 30 ms; FA = 90°; FOV = 240 × 240 mm^2^; slice thickness = 5 mm; slices = 30; matrix size = 64 × 64; and total volumes = 185. During the 6 min 10 s functional scan, subjects were instructed to keep their eyes closed, keep still, stay awake, and not to think about anything systematically. After the data acquisition, subjects were asked whether or not they remained awake during the whole procedure.

### 2.3 Structural MRI data analysis

The intracranial volume (ICV) and regional brain volumes were obtained by applying the FreeSurfer 5.0 (http://surfer.nmr.mgh.harvard.edu) longitudinal processing pipeline on the T1‐weighted MPRAGE images as described in our previous studies (Cai et al., 2016; Li et al., 2015; Yuan et al., 2013). The processing procedure consisted of (a) skull stripping; (b) automated Talairach transformation; (c) segmentation of the subcortical white matter and deep gray matter volumetric structures; (d) intensity normalization; (e) tessellation of the gray matter/white matter boundary; (f) automated topology correction; (g) surface deformation; (h) registration of the subjects' brains to a common spherical atlas.

### 2.4 Resting‐state MRI data analysis

Analysis of Functional NeuroImages software (AFNI, http://afni.nimh.nih.gov/) and FMRIB's Software Library (FSL, http://www.fmrib.ox.ac.uk/fsl/) were used for fMRI resting‐state images analysis. As described in our previous study (Yuan et al., 2016, 2017), the workflow of resting‐state MRI images preprocessing was divided into eight sections, dropping the first 5 volumes, slice‐timing correction, rigid‐body motion correction, removal skull, spatial smoothing, affine registration to the skull‐stripped structural image, spatially normalized into MNI152 template, and intensity normalization. It is inadequate to control the noise induced by head motion using nuisance regression and bandpass filtering alone (Patel et al., 2014; Power, Barnes, Snyder, Schlaggar, & Petersen, 2012). Therefore, wavelet despiking was applied for the resting‐state functional connectivity (RSFC) analyses (Patel et al., 2014). The denoising steps were as follows: (a) time series despiking (wavelet domain); (b) nuisance signal regression (14‐parameters regression); (c) a temporal Fourier filter (0.009–0.10 Hz). The regions of both left and right thalamus were chosen as our seeds, which were labeled according to Harvard‐subcortical structural atlas (http://www.cma.mgh.harvard.edu/). The regional resting‐state fMRI time series was generated by using the average functional time series of all voxels extracted for each ROI in the template atlas. To investigate the strength of RSFC between each ROI and the other voxel within the brain, Pearson correlation was carried out respectively for left thalamus and right thalamus, and then Fisher's *r*‐to‐*z* transformation was employed.

### 2.5 Statistical analysis

The demographic characteristics and the volume of both left and right thalamus were extracted and imported into the SPSS 20.0 (SPSS Statistics, IBM, Armonk, NY). Demographic characteristics were compared by using independent *t*‐tests between AD and HC. Pearson correlations were applied between the poor sleep quality measure (PSQI total score) and the alcohol dependence measure (AUDIT score). All tests were two‐tailed, and the level of significance was *p* < .05.

For each structure, the volumes for both the left and right thalamus were normalized by the brain volume and group analyses were conducted using independent two‐sample *t* tests between AD and HC. Bonferroni procedure was employed to correct for multiple comparisons. All tests were two‐tailed, and the level of significance was *p* < .025 (.05/2).

Then two‐sample *t* tests were used to compare *z* value maps between AD and HC. Permutation‐based nonparametric testing with 5,000 random permutations was used to investigate the group comparisons of functional connectivity. To control for multiple comparisons, threshold‐free cluster‐enhancement (TFCE) was used, and the significance threshold was set to *p* < .05, familywise‐error (FWE) corrected in line with current reporting guidelines (Eklund, Nichols, & Knutsson, 2016). The correlations between the neuroimaging findings (i.e., abnormal RSFC of thalamus with other regions) and behavioral data in AD (i.e., AUDIT, PSQI) were assessed by Pearson correlations. The Bonferroni correction was used in this part to correct for multiple comparisons.

We employed mediation analyses to test whether the relationship between any two correlated variables (AUDIT and PSQI score) can be explained by the values from a third variable (RSFC of thalamus with other regions) by using PROCESS bootstrapping and bias‐corrected 95% confidence intervals (CIs) for SPSS 22 (IBM, Armonk, NY; Preacher & Hayes, 2004). With this software, we calculated the direct effect of alcohol dependence on subjective sleep quality in the presence of the impairment resting‐state connectivity. We further measured the effect of alcohol dependence on mediating role of thalamus connectivity; the effect of alcohol dependence on subjective sleep quality via altered resting‐state connectivity (i.e., indirect effect); and the effect of altered resting‐state connectivity on subjective sleep quality in the presence of alcohol dependence. According to standard convention (Kober et al., 2010), “X” refers to the AUDIT score, “Y” refers to the PSQI score, and “M” refers to left thalamus–mPFC RSFC strength in current study (Yuan et al., 2016, 2017).

## 3 RESULTS

### 3.1 Demographics

Detailed demographical analysis showed there were no significant differences for age between two groups. Significant group differences were observed for multiple drinking measures such that AD patients reported higher ADS score (*t* = 3.83, *p* = .001) and higher AUDIT score (*t* = 4.60, *p* < .001). Additionally, AD exhibited higher scores in self‐reports of anxiety (*t* = 2.70, *p* = .012) and self‐reports of depression (*t* = 2.18, *p* = .038) compared to controls. However, there were no significant group differences in BIS‐11 (*t* = 2.00, *p* = .056; Table 1).

### 3.2 PSQI score difference

**Figure 1:**
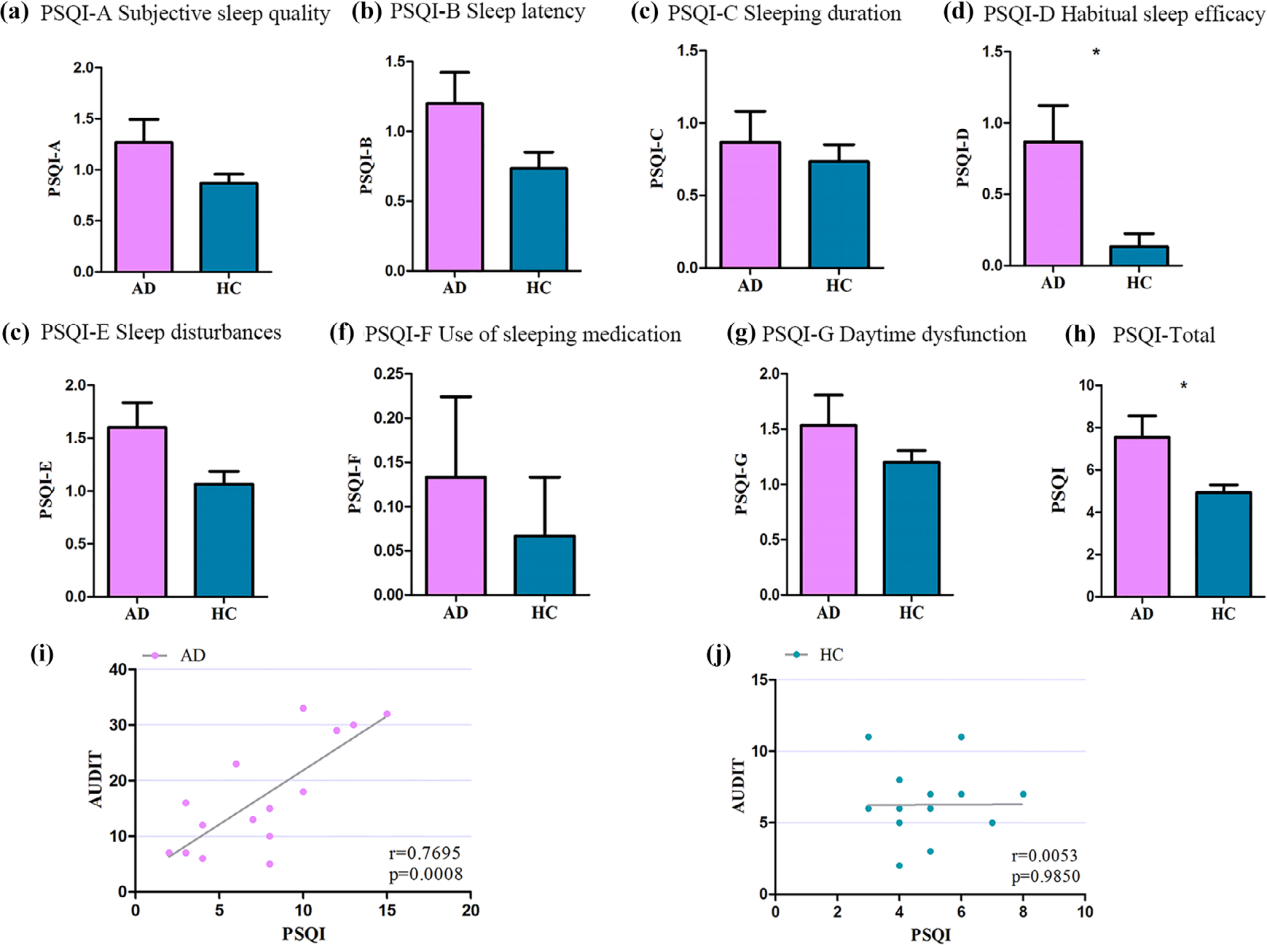
(a–h) PSQI scores in alcohol‐dependent patients and healthy controls. (i, j) The correlation between the PSQI score and the AUDIT score. Alcohol‐dependent patients committed higher scores than controls in sleep efficacy and total score. Correlation analysis revealed significant positive correlations between the PSQI and AUDIT score in alcohol‐dependent patients (i, *r* = .7695, *p* = .0008). With regard to the controls, no significant correlations were detected between PSQI score and AUDIT score [Color figure can be viewed at http://wileyonlinelibrary.com]

Individuals with AD reported significantly higher scores in Sleep efficacy (*t* = 2.70, *p* = .012) and total score (*t* = 2.41, *p* = .026; Table 1 and Figure 1) than HC.

AD showed a significant positive correlation between the sleep quality (PSQI total scores) and alcohol use disorder measure (AUDIT score; *r* = .6975, *p* = .0008).

### 3.3 Thalamus volumes differences

**Figure 2:**
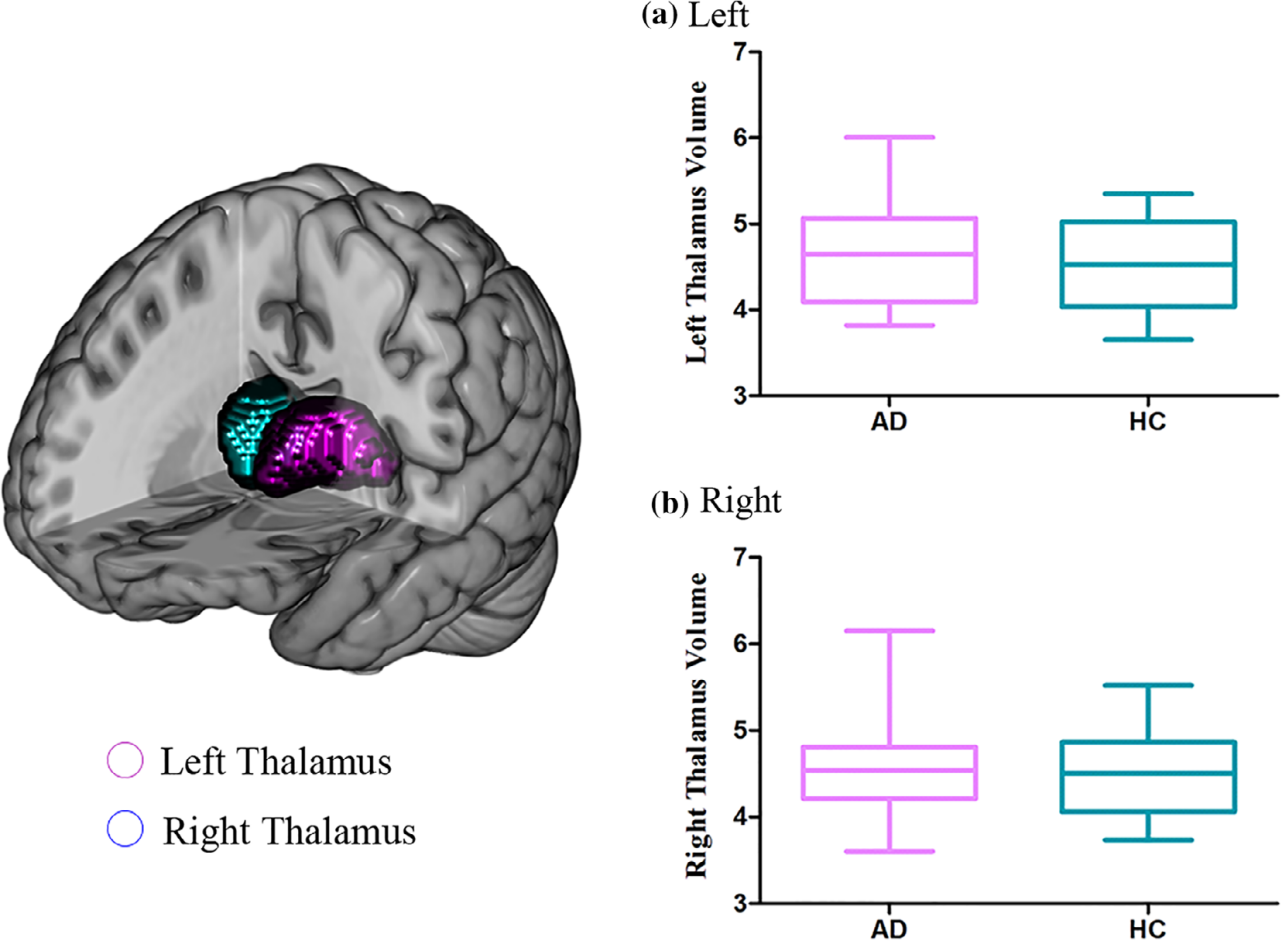
Comparisons of the thalamus volumes between alcohol‐dependent patients and controls. No significant group effect on the normalized left thalamus and right thalamus [Color figure can be viewed at http://wileyonlinelibrary.com]

Two‐sample *t* tests indicated that there was no significant group difference in volume for the normalized left thalamus (*t* = 0.67, *p* = .50) and right thalamus (*t* = 0.37, *p* = .071; Figure 2 and Table 2).

**Table 2:** Normalized volume of regions comparison between AD and HC

Region	Volume (mean ± SD)		*t* value	*p*‐value
	AD (*n* = 15)	HC (*n* = 15)		
Left				
Thalamus	4.62 ± 0.62	4.48 ± 0.51	0.678	.504
Right				
Thalamus	4.57 ± 0.59	4.49 ± 0.54	0.367	.717

Values are mean ± SD unless otherwise indicated.

### 3.4 RFSC differences

**Figure 3:**
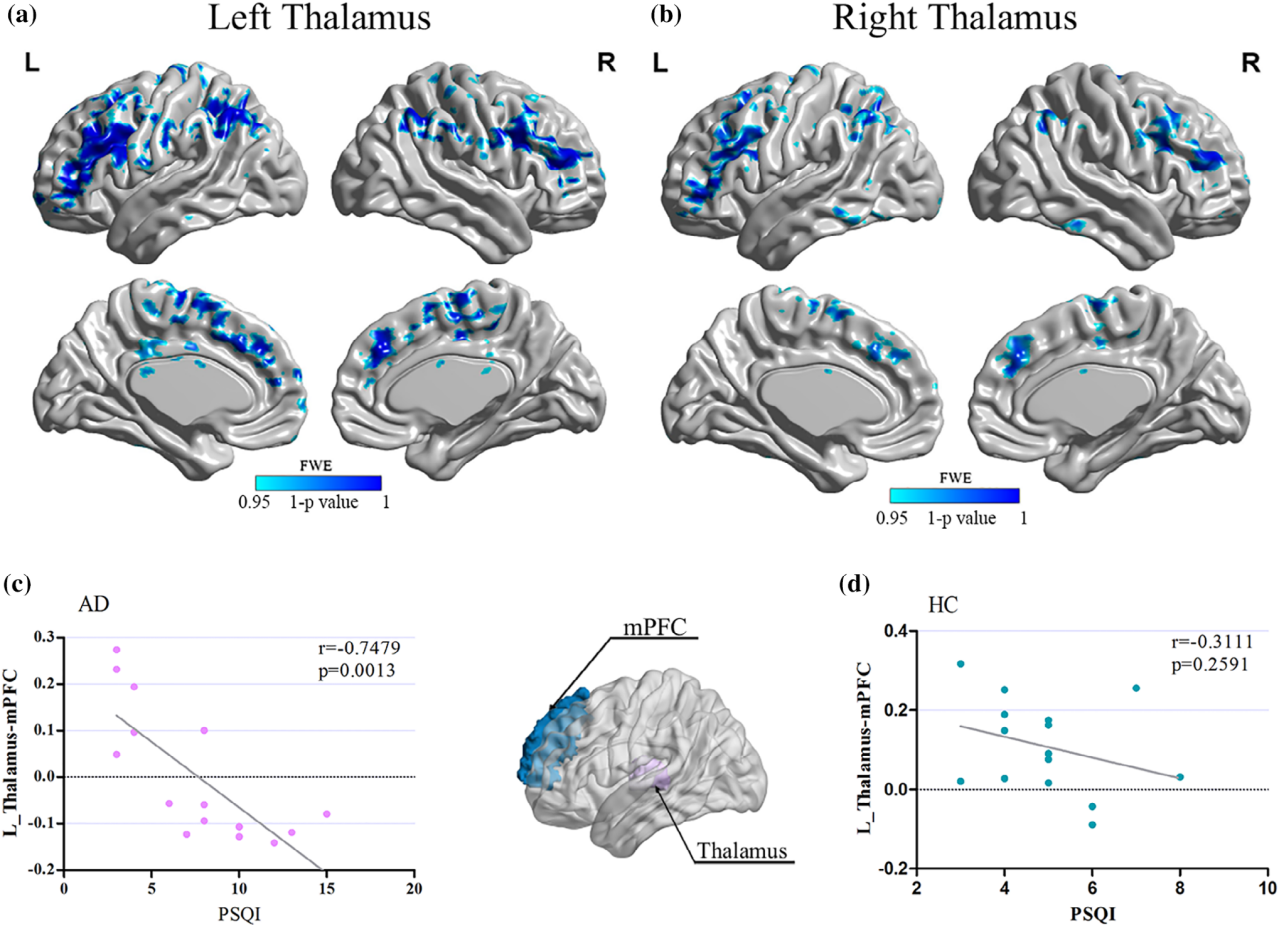
(a, b) The resting‐state functional connectivity (RSFC) of thalamic differences between alcohol‐dependent patients and controls. (c, d) The correlation between functional connectivity (medial prefrontal cortex–thalamus) and PSQI score. Significantly lower resting‐state functional connectivity (RSFC) showed between left thalamus and several regions in alcohol‐dependent patients (a, FWE corrected, *p* < .05), that is, bilateral medial prefrontal cortex (mPFC), orbitofrontal cortex (OFC), anterior cingulate cortex (ACC), left angular gyrus, and right caudate. In addition, the right thalamus exhibited decreased RSFC with bilateral mPFC, OFC, and left caudate in alcohol‐dependent patients (b, FWE corrected, *p* < .05). Significant negative correlation (*r* = .7479; *p* = .0013) was found between the left medial prefrontal cortex–thalamus RSFC strength and PSQI score in alcohol‐dependent patients (c) [Color figure can be viewed at http://wileyonlinelibrary.com]

RSFC analysis generated similar left and right thalamus networks in AD and HC, including the cortical regions (OFC, ACC, angular, parietal, occipital, temporal, and limbic cortices), subcortical regions (caudate), and cerebellum. However, further analysis revealed reduced RSFC between left thalamus and several regions in AD (*p* < .05, FWE corrected), that is, bilateral medial prefrontal cortex (mPFC), orbitofrontal cortex (OFC), anterior cingulate cortex (ACC), left angular gyrus, and right caudate (Table 3 and Figure 3a). In addition, the right thalamus exhibited decreased RSFC with bilateral mPFC, OFC, and left caudate in AD group (Table 3 and Figure 3b).

**Table 3:** Regions exhibiting significantly decreased RSFC with left thalamus between AD and HC

Region	Brodmann area	Peak voxel		Volume (mm^3^)		Peak *p*‐value
		*X*	*Y*	*Z*		
Regions showed decreased RSFC with left thalamus in AD relative to HC						
Left medial superior frontal gyrus	8/9	−8	40	36	2,360	.001
Right medial superior frontal gyrus	8/9	4	40	36	2,024	.001
Left orbitofrontal gyrus	10/11/47	−46	40	−2	1,472	.0006
Right orbitofrontal gyrus	10/11/47	36	36	−2	272	.0086
Left anterior cingulate gyrus	32	−14	46	14	336	.0062
Right anterior cingulate gyrus	32/33	18	22	28	600	.0016
Left angular gyrus	39	−36	−60	32	584	.0002
Right caudate	25	18	8	26	432	.0006
Regions showed decreased RSFC with right thalamus in AD relative to HC						
Left medial superior frontal gyrus	8/9	2	40	30	280	.0078
Right medial superior frontal gyrus	8/9	8	40	41	512	.0112
Left orbitofrontal gyrus	10/11/47	−41	41	−2	616	.0026
Right orbitofrontal gyrus	10/11/47	40	40	−2	184	.0156
Left caudate	25	−10	−4	21	112	.021

All the coordinates are located in the MNI space.

Abbreviation: RSFC: resting‐state functional connectivity.

### 3.5 Brain–behavior correlation results

Pearson correlation was applied between the 8 and 5 regions that showed abnormal RSFC with left and right thalamus, respectively, and clinical variables (i.e., AUDIT, PSQI). All tests were two‐tailed, and to correct for multiple comparisons we used Bonferroni correction (*p* < .0019 [.05/26]). According to these criteria, the PSQI score was negatively correlated with the RSFC strength between the left thalamus and ipsilateral medial prefrontal cortex in AD (*r* = .7479; *p* = .0013; Figure 3c). Correlation analysis revealed significant negative correlations between RSFC strength of left thalamus–mPFC and AUDIT score (*r* = −.64; *p* = .01). Additionally, several other correlations were significant (*p* < .05), but did not survive correction for multiple comparisons (*p* < .0019). There was no significant correlation between the RSFC strength of left thalamus–mPFC and SAS, SDS, or BIS‐11 scores. No other significant results were detected between the volume of both left and right thalamus and behavioral data.

### 3.6 Mediation analysis

**Figure 4:**
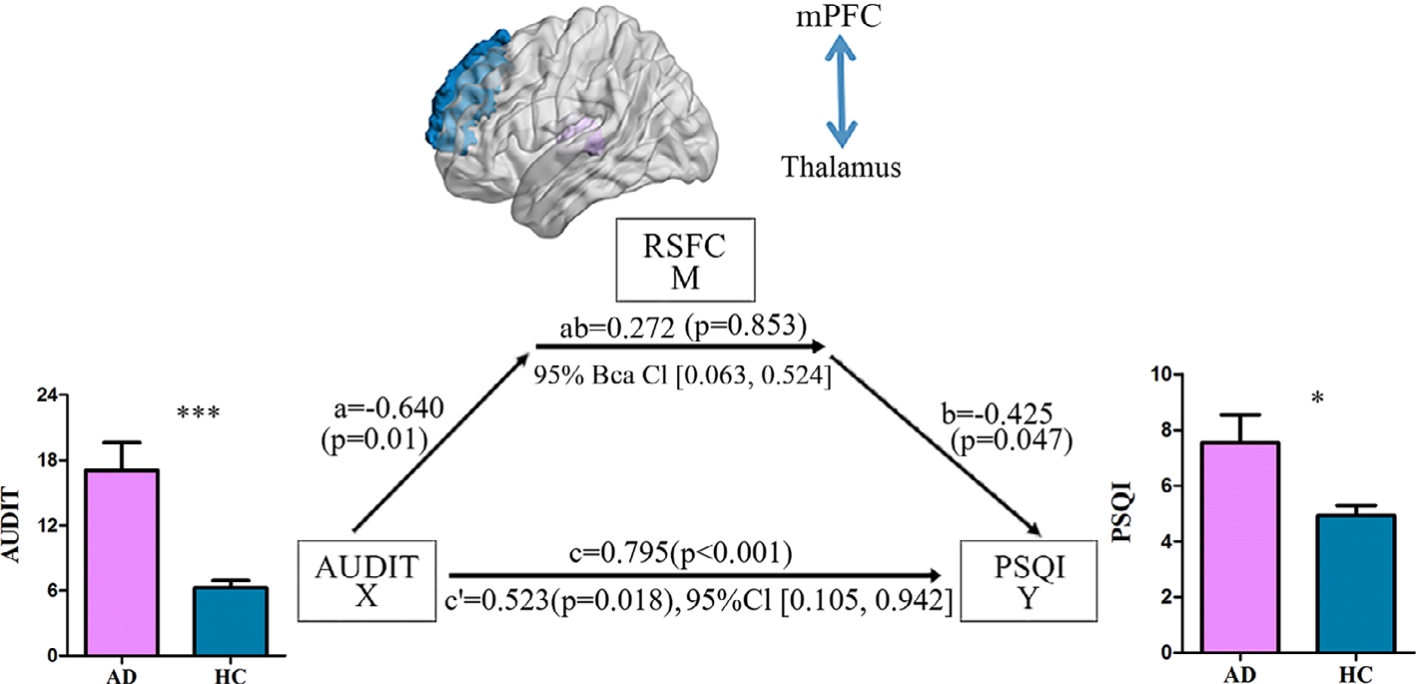
Mediation analysis. The left thalamus–mPFC *z* value (i.e., thalamus–mPFC) mediated the relationship between AUDIT score and PSQI score in alcohol‐dependent patients (*a* × *b* = 0.272, *p* = .853; *c*′ = 0.523, *p* = .018) [Color figure can be viewed at http://wileyonlinelibrary.com]

Given the finding that PSQI score was correlated with left thalamus–mPFC RSFC strength in AD, together with the correlation with AUDIT score, mediation modeling was used to test whether the magnitude of alcohol dependence effect on sleep impairment depended on impaired left thalamus–mPFC RSFC (Hayes, 2012). Mediation analysis was applied among AUDIT score, PSQI score, and left thalamus–mPFC RSFC strength in AD. As expected, we revealed that the RSFC strength of the left thalamus–mPFC pathway partly mediated the relationship between AUDIT score and PSQI score (*a* × *b* = 0.272, *p* = .853; *c*′ = 0.523, *p* < .001; Figure 4). No other regions' connectivity with left thalamus was significant mediators of AUDIT‐PSQI associations.

## 4 DISCUSSION

We observed that the RSFC of left thalamus–mPFC with PSQI score, which provided novel insights into the alcohol dependence—sleep impairments associations in abstinent males. We also for the first time to date, formally demonstrate that the RSFC of left thalamus–mPFC played a mediating role in the association of alcohol dependence and poor sleep quality. We computed the connectivity strength and showed in mediation analyses that problem drinking is related to sleep impairment via decreased thalamocortical connectivity. This study may provide new insight for sleep impairment in male AD patients, and this in turn has implications for treatment because of the brain areas identified.

### 4.1 Sleep impairments in AD patients

In this study, we revealed the poor subjective sleep quality in AD patients (Figure 1). The association between AUDIT score and PSQI score in AD patients was also found the chronic effect of alcohol on the sleep impairment among drinkers. The relationship between alcohol dependence and sleep impairments is of great complexity. While acute alcohol intake in nonalcoholic social drinkers reduces sleep onset latency and improves NREM sleep (Roehrs & Roth, 2012), alcohol dependence is generally associated with sleep impairments (Thakkar et al., 2015) although the data vary widely across studies (Chakravorty et al., 2016; Hartwell, Bujarski, Glasner‐Edwards, & Ray, 2015; Zhabenko et al., 2012). On the other hand, alcohol causes adenosine buildup to disrupt sleep homeostasis. Adenosine can inhibit wake–active neurons via adenosine A_1_ receptor thus disinhibiting sleep–active neurons, which also stimulates sleep–active neurons via A_2A_ receptors (Alam & McGinty, 2017). In addition, adenosine is a key mediator of neuronal responses to alcohol (Newton & Messing, 2006). This property permits alcohol to have a secondary effect on virtually all neurotransmitter/neuromodulator systems related to homeostatic regulation of sleep within the brain. Therefore, it follows that alcohol has great impact on sleep. Taken together, by revealing AD patients had a greater association between AUDIT score and PSQI score than controls, we provided partial evidence for the sleep impairment in AD patients.

### 4.2 RSFC of the left thalamus to mPFC mediates the relationship between alcohol dependence and sleep impairments in AD patients

We noticed that AUDIT score was correlated with PSQI score only in AD patients (Figure 1). Moreover, the left thalamus–mPFC RSFC strength was correlated with PSQI score as well as AUDIT score in AD patients (Figure 3). We suggested that thalamocortical circuits RSFC strength could be a biomarker of sleep quality and alcohol addiction severity. We proposed that thalamocortical RSFC (i.e., thalamus–mPFC) mediated the relationship between AUDIT score and PSQI score, which was confirmed by our mediation analysis results (Figure 4). We suggest that alcohol drinking regulated the sleep quality via the reduced thalamocortical RSFC in AD patients.

The thalamus is critical in sleep homeostasis through its regulation by adenosine to its wake‐promoting cholinergic neurons innervating the basal forebrain (Brown, Basheer, McKenna, Strecker, & McCarley, 2012). Adenosine A1 receptors are upregulated in the PFC, which may consequently produce inhibition of PFC activity (Elmenhorst et al., 2007). These findings may support the theory that alcohol, via its action on adenosine uptake, increases extracellular adenosine resulting in the inhibition of wake‐promoting neurons from thalamus to basal forebrain and the activity of PFC (Kalinchuk, McCarley, Porkka‐Heiskanen, & Basheer, 2011; Thakkar et al., 2015).

### 4.3 Reduced RSFC with cortico‐striato‐thalamo‐cortical loop circuits in AD patients

We also provided evidence for the role of the thalamus‐centered circuits in alcohol dependence. Anatomically, thalamus receives projections from multiple cortical and midbrain regions, which is integral to the circuits that underlie the reward (Haber & Calzavara, 2009) and response inhibition processes mediating control processes in goal‐oriented behaviors (de Bourbon‐Teles et al., 2014; Phillips, Kambi, & Saalmann, 2016). Previous neuroimaging studies observed structural and functional differences of thalamus between substance use disorder and healthy controls (Feil, Sheppard, Fitzgerald, Yücel, & Lubman, 2010). Several studies showed that reduced thalamic connectivity was associated with increased craving and other measures of severity of drug dependence (Liao et al., 2016; Wang et al., 2017). However, the implication of thalamocortical circuits in neural mechanisms of AD patients remains unclear. Our findings contribute toward filling this gap by showing the reduced RSFC between thalamus and a series of regions in AD patients (Figure 3), such as mPFC (involved in cognitive control), OFC (involved in goal‐directed behaviors), ACC (involved in inhibitory control and awareness), and caudate (involved in reward; Beck et al., 2012; Bruno & Sakmann, 2006; Cai et al., 2016; Li et al., 2015; Yuan et al., 2013, 2016; Yuan, Yu, Cai, et al., 2017). Tract tracing studies in nonhuman primates have determined that the striatum projects via the globus pallidus to the thalamus, which then projects back to the frontal cortex (Haber & McFarland, 2001). The thalamus functions as both input and output structures within cortico‐striato‐thalamo‐cortical loop circuits (Huang, Mitchell, Haber, Alia‐Klein, & Goldstein, 2018). Evidently, accurate roles of this circuit should be investigated in the future by employing the more sensitive method and nuanced experimental design.

In the current study, we assessed the possible mediator role of right thalamus center‐circuits in the relationship between AUDIT and PSQI in AD patients. We observed no significant results in the right thalamus. The absence of right thalamus involvement in our findings may be attributable the small sample. Larger and longitudinal studies should be implemented in future to assess the effects of thalamus on sleep impairment in alcohol dependence patients. Evidently, the predominant left lateralization of the findings in the current study should be verified in the future by larger sample size.

### 4.4 Limitation

Several limitations are considered. First, we only employed subjective measures of sleep in this study. Previous experiments associating PSQI with resting‐state experiments also failed to measure objective measures of sleep (Klumpp et al., 2017; Klumpp, Hosseini, & Phan, 2018). Objective measures, such as polysomnography should be used to further investigate the association of alcohol dependence and sleep in future study. Second, our sample was relatively small, and therefore our group comparisons were not powered to detect small effects. Third, the thalamus is a very complex brain region and has distinctive subregions with different functions; accordingly, RSFC in distinctive subregions of thalamus in AD could be taken into consideration in future studies. Additionally, we cannot extend our findings to females with AD as our sample included only males. Due to the lack of longitudinal experiment design, we failed to show whether the sleep disorder came before alcohol dependence or the other way around. In the current study, we focused on the sleep impairments in AD patients. Still, we agree that the associations of PSQI and anxiety and depression in the AD patients should be investigated in future work. Recently, Dopamine D2 receptors has been implicated in sleep deprivation, and in alcohol and other substance use disorders (Volkow et al., 2008, 2012; Wiers et al., 2016). In the current study, we did not collect objective sleep measurements for the night before the scanning. Thus, we cannot exclude the possible acute effects of sleep on our resting state results.

## 5 CONCLUSION

We revealed reduced RSFC within cortico‐striato‐thalamo‐cortical loops that were correlated with drinking behavior and sleep impairments in abstinent male AD patients. More importantly, the left thalamus–mPFC RSFC strength mediated the relationship between the AUDIT score and PSQI score in individuals with alcohol dependence. It is hoped that our findings may shed new insights into the neural mechanisms of sleep and compulsive alcohol use. This study may provide new information for sleep impairment treatment in individuals with alcohol dependence.

## Data Availability

The data that support the findings of this study are available from the corresponding author upon reasonable request.
